# Supplementary material for: Levels of Knowledge, Beliefs, and Practices Regarding Osteoporosis and the Associations with Bone Mineral Density among Populations More Than 40 Years Old in Malaysia
Source: Int J Environ Res Public Health. 2019 Oct 25;16(21):4115. doi: 10.3390/ijerph16214115 (PMC6861980; doi:10.3390/ijerph16214115)
Supplement: Supplementary file 1 [file ijerph-16-04115-s001.pdf]

Table S1. The distribution of responses to osteoporosis knowledge questions of subjects

Items	Correct Ans	Overall (N=786)			Men (n=382)			Women (n=404)			Correct responses (N=786)	
		T	F	DK	T	F	DK	T	F	DK	n	%
1. Makes bone become brittle and weak from loss of tissue, thus more likely to break (fracture).	T	753 (95.8)	19 (2.4)	14 (1.8)	362 (94.8)	11 (2.9)	9 (2.4)	391 (96.8)	8 (2.0)	5 (1.2)	753	95.8
2. Osteoporosis will result in knee pain.	F	509 (64.8)	166 (21.1)	111 (14.1)	232 (60.7)	82 (21.5)	68 (17.8)	277 (68.6)	84 (20.8)	43 (10.6)	166	21.1
3. Postmenopausal women have no risk to develop osteoporosis.	F	20 (2.5)	619 (78.8)	147 (18.7)	15 (3.9)	248 (64.9)	119 (31.2)	5 (1.2)	371 (91.8)	28 (6.9)	619	78.8
4. Osteoporosis is an untreatable disease.	F	167 (21.2)	513 (65.3)	106 (13.5)	79 (20.7)	256 (67.0)	47 (12.3)	88 (21.8)	257 (63.6)	59 (14.6)	513	65.3
5. Osteoporosis can be diagnosed by measuring bone mineral density.	T	703 (89.4)	12 (1.5)	71 (9.0)	330 (86.4)	7 (1.8)	45 (11.8)	373 (92.3)	5 (1.2)	26 (6.4)	703	89.4
6. I do not need to do bone mineral density test unless I fracture my bones.	F	94 (12.0)	673 (85.6)	19 (2.4)	62 (16.2)	309 (80.9)	11 (2.9)	32 (7.9)	364 (90.1)	8 (2.0)	673	85.6
7. Consume calcium supplements help me to prevent osteoporosis.	T	679 (86.4)	58 (7.4)	49 (6.2)	334 (87.4)	24 (6.3)	24 (6.3)	345 (85.4)	34 (8.4)	25 (6.2)	679	86.4
8. The regular intake of calcium supplements can lead to formation of kidney stones.	F	413 (52.5)	108 (13.7)	265 (33.7)	195 (51.0)	47 (12.3)	140 (36.6)	218 (54.0)	61 (15.1)	125 (30.9)	108	13.7

9. Foods such as milk, tofu, yellow dhal and spinach are rich in calcium.	**T**	733 (93.3)	13 (1.7)	40 (5.1)	346 (90.6)	8 (2.1)	28 (7.3)	387 (95.8)	5 (1.2)	12 (3.0)	733	93.3
10. By exposing my skin to sunlight for about 15 minutes a day, I can obtain my recommended daily intake of vitamin D.	**T**	460 (58.5)	217 (27.6)	109 (13.9)	239 (62.6)	91 (23.8)	52 (13.6)	221 (54.7)	126 (31.2)	57 (14.1)	460	58.5
11. Intake of glucocorticoids may increase the risk of osteoporosis.	**T**	330 (42.0)	50 (6.4)	406 (51.7)	156 (40.8)	24 (6.3)	202 (52.9)	174 (43.1)	26 (6.4)	204 (50.5)	330	42.0
12. I must have good vision and comfortable shoes with good grip to prevent me from falling.	**T**	737 (93.8)	44 (5.6)	5 (0.6)	359 (94.0)	21 (5.5)	2 (0.5)	378 (93.6)	23 (5.7)	3 (0.7)	737	93.8

**T* True, *F* False, *DK* Don't Know

Table S2. Response to the OHBS among the subjects

Items	Health Beliefs scale, N (%)														
	Overall (N=786)					Men (n=382)					Women (n=404)				
	SD	D	N	A	SA	SD	D	N	A	SA	SD	D	N	A	SA
1. You have high chance to get osteoporosis.	9 (1.1)	212 (27.0)	189 (24.0)	335 (42.6)	41 (5.2)	8 (2.1)	132 (34.6)	93 (24.3)	135 (35.3)	14 (3.7)	1 (0.2)	80 (19.8)	96 (23.8)	200 (49.5)	27 (6.7)
2. You more likely to get osteoporosis because of your family history.	16 (2.0)	507 (64.5)	89 (11.3)	163 (20.7)	11 (1.4)	11 (2.9)	242 (63.4)	38 (9.9)	86 (22.5)	5 (1.3)	5 (1.2)	265 (65.6)	51 (12.6)	77 (19.1)	6 (1.5)
3. It would be very serious if you got osteoporosis.	3 (0.4)	134 (17.0)	96 (12.2)	444 (56.5)	109 (13.9)	2 (0.5)	62 (16.2)	45 (11.8)	211 (55.2)	62 (16.2)	1 (0.2)	72 (17.8)	51 (12.6)	233 (57.7)	47 (11.6)
4. Exercise regularly helps to build strong bones.	2 (0.3)	33 (4.2)	42 (5.3)	575 (73.2)	134 (17.0)	1 (0.3)	16 (4.2)	18 (4.7)	262 (68.6)	85 (22.3)	1 (0.2)	17 (4.2)	24 (5.9)	313 (77.5)	49 (12.1)
5. Intake of enough calcium prevents you from getting osteoporosis.	-	42 (5.3)	62 (7.9)	600 (76.3)	82 (10.4)	-	21 (5.5)	30 (7.9)	287 (75.1)	44 (11.5)	-	21 (5.2)	32 (7.9)	313 (77.5)	38 (9.4)
6. You feel that you are not strong enough to exercise regularly.	37 (4.7)	477 (60.7)	81 (10.3)	188 (23.9)	3 (0.4)	29 (7.6)	257 (67.3)	23 (6.0)	71 (18.6)	2 (0.5)	8 (2.0)	220 (54.5)	58 (14.4)	117 (29.0)	1 (0.2)
7. Starting a new habit to exercise regularly is hard for you to do.	29 (3.7)	476 (60.6)	105 (13.4)	173 (22.0)	3 (0.4)	24 (6.3)	254 (66.5)	37 (9.7)	65 (17.0)	2 (0.5)	5 (1.2)	222 (55.0)	68 (16.8)	108 (26.7)	1 (0.2)
8. Calcium-rich foods are very costly.	11 (1.4)	587 (74.7)	83 (10.6)	98 (12.5)	7 (0.9)	3 (0.8)	272 (71.2)	50 (13.1)	53 (13.9)	4 (1.0)	8 (2.0)	315 (78.0)	33 (8.2)	45 (11.1)	3 (0.7)
9. You do not like calcium-	36	645	86	18 (2.3)	1 (0.1)	18 (4.7)	305	46	12 (3.1)	1 (0.3)	-	18 (4.5)	340	40 (9.9)	6 (1.5)

rich foods.	(4.6)	(82.1)	(10.9)				(79.8)	(12.0)					(84.2)		
10. You always look for new information related to health.	-	78 (9.9)	112 (14.2)	548 (69.7)	48 (6.1)	-	48 (12.6)	53 (13.9)	255 (66.8)	26 (6.8)	-	30 (7.4)	59 (14.6)	293 (72.5)	22 (5.4)
11. Even you are not sick, but you will do regular health check-up.	-	190 (24.2)	72 (9.2)	483 (61.5)	41 (5.2)	-	95 (24.9)	31 (8.1)	235 (61.5)	21 (5.5)	-	95 (23.5)	41 (10.1)	248 (61.4)	20 (5.0)
12. You will follow recommendations to keep you healthy.	1 (0.1)	9 (1.1)	65 (8.3)	646 (82.2)	65 (8.3)	-	6 (1.6)	29 (7.6)	312 (81.7)	35 (9.2)	1 (0.2)	3 (0.7)	36 (8.9)	334 (82.7)	30 (7.4)

**SD* Strongly Agree, *D* Disagree, *N* Neutral, *A* Agree, *SA* Strongly Agree
